# Clinical features and prognosis of transient global amnesia in Israel: 6 years’ single-center experience

**DOI:** 10.1007/s13760-024-02703-w

**Published:** 2024-12-30

**Authors:** Ido Gindes, Itzhak Kimiagar, Shlomi Peretz, Gilad Kenan

**Affiliations:** 1 Tel-Aviv university, Tel Aviv-Yafo, Israel; 2 Department of Neurology, Shamir Medical Center, Be`er Ya`akov, Israel

**Keywords:** TGA, Transient global amnesia, Amnesia, Epidemiology, Pathogenesis, Incidence, Israel

## Abstract

**Background:**

Transient global amnesia (TGA) is a benign neurological syndrome of unknown etiology, causing sudden anterograde amnesia that lasts up to 24 h. During the episode of TGA, other cognitive functions are normal. This is the first study describing the characteristics of the disease in Israel.

**Methods:**

A retrospective review of all patients with a confirmed diagnosis of TGA at Shamir Medical Center (SMC) between January 2016 and December 2021.

**Results:**

One-hundred and four patients with confirmed TGA were identified, with an average age of 64 years (range: 39–87). The annual incidence of TGA was 2.52 per 100,000 and 6.96 per 100,000 among those over 50, with a slight female predominance. The recurrence rate was 11.5%. 61.5% of patients had one or more cerebrovascular risk factors. A precipitating factor was described in 30.8% of cases, with emotional stress, Valsalva maneuver and sexual intercourse being the most prevalent. Chronic ischemic changes were the most common imaging abnormality. Focal slowing was found in nine patients, and epileptic activity was found in four patients.

**Conclusions:**

In this study, we showed that patient characteristics and clinical features of TGA patients in Israel were similar to those described in other reports worldwide. We were unable to demonstrate a specific risk factor or a causative agent. Interestingly, the annual incidence in Israel was lower than in other countries.

## Introduction

Transient global amnesia (TGA) is characterized by the sudden onset of anterograde amnesia, that typically lasts few hours and by definition resolves within 24 h, with otherwise normal neurological examination. Laboratory tests, computed tomography (CT) scans and electroencephalogram (EEG) results are typically normal [1–4, 8]. However, such investigations are usually necessary to exclude alternative diagnoses such as encephalitis, stroke, transient ischemic attack, metabolic disorders such as hypoglycemia or a focal seizure (including transient epileptic amnesia) [1, 19]. 

In some patients, the TGA attack may be triggered by the Valsalva maneuver, emotional stress, immersion in cold or hot water, sexual intercourse, pain, physical exertion, and medical procedures. However, in most cases, no clear precipitating factor is identified [1–5]. 

The annual incidence of TGA worldwide has been reported to be 3 to 10.4 per 100,000 persons in the whole population and 23.5 to 32 per 100,000 persons among those aged 50 years or more [1–4, 6]. Few TGA patients (2.9–25%) will experience a second episode [1, 4, 8].

The causes and mechanisms of TGA are unknown [4]. The current theories suggest three potential pathogeneses: vascular, due to venous flow disturbances or focal arterial ischemia; [9, 12–15] epileptic; [16] and migraine related [17, 18]. 

No study has been conducted to evaluate the clinical features of patients suffering from TGA in Israel. In this study, we set out to evaluate the epidemiological, clinical, and prognostic features of patients with TGA in Shamir Medical Center (SMC) during a 6-year period. Our findings will allow patients and physicians to better understand disease prevalence, risk factors, and outcomes and improve patient counseling.

## Methods

All potential cases of TGA in SMC between January 2016 and December 2021 were retrospectively identified based on a coded diagnosis of Transient Global Amnesia (ICD-9 code 437.7) on their admission or discharge report. We reviewed neurological consultations, admission records, and discharge summaries to extract epidemiological data, as well as information about potential precipitating factors and ancillary test results. The variables were analyzed using T-square, chi-square, and Z-score tests. A significance level of 0.05 has been used to determine the statistical significance of the results. Incidence was calculated using a government database’s estimation that SMC serves a population of 700,000.

### Inclusion criteria

Adult patients with first-time or recurrent TGA episodes fulfilling the diagnostic criteria suggested by Caplan [20] and further consolidated by Hodges and Warlow [5]: (1) A witnessed attack with information available from a capable observer who was present for most of the attack; (2) Anterograde amnesia during the attack; (3) Cognitive impairment limited to amnesia without clouding of consciousness or loss of personal identity; (4) Absence of accompanying focal neurological symptoms during the attack and significant neurological signs afterward; (5) Absence of epileptic features; and (6) Resolution of attacks within 24 h.

Patients with an alternative diagnosis better explaining the symptoms were excluded.

## Standard workup of patients with TGA

At our hospital, the standard initial evaluations of TGA typically include routine blood tests, brain CT, carotid ultrasound, and Holter ECG monitoring. While MRI is rarely performed during hospitalization, it is often recommended post-discharge to investigate potential structural causes. EEG is usually performed, especially when epileptic aetiology is suspected. Additional tests are guided by the patient’s symptoms and the clinical judgment of the attending physician.

## Ethics

This study was conducted in accordance with the principles outlined in the Declaration of Helsinki. The study protocol was reviewed and approved by the Shamir Medical Center Institutional Review Board, which waived the requirement for informed consent due to the retrospective nature of the research.

## Results

129 patients were identified with potential TGA based on their coded diagnosis. Twenty-five patients were excluded: 21 for not fulfilling the diagnostic criteria of TGA; and 3 had an alternative diagnosis better explaining the symptoms, including two cases of transient ischemic attack (TIA) and one case of stroke. Consequently, 104 patients were included in the final analysis. Table 1 presents their epidemiological features, cerebrovascular risk factors and inpatient and outpatient evaluations.

**Table 1:** Epidemiological features, cerebrovascular risk factors, inpatient and outpatient evaluation of TGA patients

Epidemiology	
Number of patients	104
Male/female (%female)	38/66 (63.5%)
Average age (years) at onset of the first episode (range)	64.4 (39–87)
Annual incidence (per 100,000 persons)	2.52
Annual incidence over the age of 50 (per 100,000 persons)	6.96
Hospitalization duration (days)	3.6 ± 1.2
Identified precipitating factor	32 (30.8%)
Identified one or more cerebrovascular risk factors	64 (61.5%)
TGA recurrence	12 (11.5%)
Headache during TGA episode	14 (13.5%)
Cerebrovascular Risk Factors*	
Hypertention	42 (40.4%)
Dyslipidemia	41 (39.4%)
Diabetes mellitus	11 (10.6%)
Smoking	11 (10.6%)
Ischemic heart disease	11 (10.6%)
Prior CVA / TIA	4 (3.8%)
**Inpatient Evaluation**	
Non-contrast computed tomography (CT)	104 (100%)
Electroencephalography (EEG)	94 (90.4%)
Carotid ultrasound	92 (88.5%)
24-hours holter electrocardiography	79 (76%)
CT-angiography	22 (21.2%)
Lumbar puncture	10 (9.6%)
**Outpatient Evaluation**	
Magnetic resonance imaging	43 (41.3%)

TGA = transient global amnesia; CVA = cerebrovascular accident; TIA = transient ischemic attack. * Some patients had multiple cerebrovascular risk factors

## Epidemiology

The average age for patients with TGA was 64 years and spanned from 39 to 87 years-old. The incidence of TGA was found to be higher in females (p-value = 0.02), but male patients tended to develop the syndrome at an earlier age, with a mean age of 62 ± 8.7 years compared to 65.9 ± 7.1 years in females (p-value = 0.02). Most TGA episodes lasted few hours (range: 10 min to 24 h) in both male and female patients. Although TGA appeared to be more prevalent in autumn and winter (58 patients) compared to spring and summer (46 patients), this difference was not statistically significant (p-value = 0.23). Most TGA patients (61.5%) had at least one cerebrovascular risk factor, as detailed in Table 1. The recurrence rate of TGA was 11.5%, with an average interval of 5.1 ± 3.7 years between episodes (1–13 years). Among the twelve patients who experienced a recurrence, three had two recurrent episodes, with an average interval of 4.7 ± 3.8 between the second and third episodes (2–9 years).

### Precipitating factors

In most patients (69.2%) no possible precipitating factor for the TGA episode was apparent. The precipitating factors of TGA patients in this study are detailed in Table 2. While some precipitating factors were more common in males and others in females, there were no statistically significant differences.

**Table 2:** Precipitating factors of TGA patients

Precipitating Factor^1^	No. of Patients
Emotional stress	12 (11.5%)
Valsalva maneuver^2^	7 (6.7%)
Sexual intercourse	6 (5.8%)
Medical procedure^3^	3 (2.9%)
New medications^4^	3 (2.9%)
Physical exertion	2 (1.9%)
Immersion in water	2 (1.9%)
Severe pain before TGA started	1 (1%)
Alcohol use^5^	1 (1%)

TGA = transient global amnesia. 1 Some patients had multiple precipitating factors.2 We included patients that described vomiting and straining to defecate before TGA started.3 Less than 24 hours before the episode.4 Including (1) Clonazepam 0.5 mg; (2) non-steroidal anti-inflammatory drug and (3) foodadditive (the name of the product or its components was unavailable).5 Less than 48 hours before the episode

## Ancillary tests

Table 1 describes the tests that were done both during hospitalization and subsequently as an outpatient. All patients had a CT scan shortly after arrival. 81.7% of patients had a normal CT scan and 14 patients (13.5%) had chronic ischemic changes. 43 patients (41.3%) had an MRI scan (all as outpatients), 48.8% of them had normal results, and 44.2% had chronic ischemic changes. None had an acute stroke or any lesion that showed restricted diffusion. A total of 94 patients (90.4%) underwent EEG, with an average time to EEG after TGA of 1.7 ± 1.1 days. 85.1% of the EEG results were normal. There were nine patients (8.7%) with focal slowings: seven were found only on the left side, one was found only on the right side, and one was bilateral (but more pronounced on the left side). Epileptiform activity was present in four patients (3.8%), three on the left and one on the right. One patient (1%) had generalized slowing.

## Discussion

In this study, we evaluated the characteristics of TGA patients in Israel for the first time. Overall, the epidemiological, clinical, and prognostic features observed were consistent with those reported globally. Ancillary testing, including brain CT, MRI, carotid ultrasound, and EEG, was generally unremarkable. In the few cases where abnormalities were identified, they were deemed incidental and unrelated to the TGA episode. In the following discussion, we address several key aspects highlighted by our findings.

### TGA features in Israel

Similar to other reports in the world, we found a slight female predominance, an increasing incidence of TGA with age and a similar recurrence rate [1, 4, 6, 7]. Based on these findings, it can be assumed that age-related factors play a role in the pathogenesis of the disease. Compared to other reports in the world [1, 4, 6], the incidence of TGA in Israel was lower.

As the exact mechanism and pathogenesis of TGA are unknown, it is difficult to determine what causes this low annual incidence. It is unlikely that this finding is due to a difference in diagnostic practices, since most studies on this syndrome use the same diagnostic criteria [5, 20]. It is also unlikely that this finding is related to poor healthcare access, since Israel has a very accessible and affordable healthcare system [21]. Potential explanations for this finding may include differences in genetics or lifestyle, as well as environmental and dietary factors.

Since migraines are a possible mechanism for TGA [17, 18], one might expect that the low incidence of TGA in Israel is correlated with a low prevalence of migraines. Surprisingly, Israel has a high migraine prevalence of approximately 18–19%, (compared to an estimated global prevalence of 14–15%) [22, 23]. This finding weakens the probability of migraine being the mechanism behind TGA.

Considering the high incidence of cerebrovascular disease risk factors (61.5%), TGA may seem to be a result of transient cerebral ischemia. However, when compared to the elderly Israeli population, there were no statistically significant differences in traditional cerebrovascular risk factors like hypertension and dyslipidemia. Moreover, TGA patients had a statistically significant lower incidence of diabetes mellitus than the general elderly population (p-value = 0.01). Consequently, the hypothesis that artery-to-artery emboli from atherosclerotic plaques cause TGA, suggested by some studies [14, 15], is not supported by these findings. Other recent studies have reached similar conclusions [11, 12]. 

### Precipitating factors: cause or association?

In our study, most patients did not have a precipitating or trigger event (69.2%). This finding is consistent with other reports worldwide (61–67%).^2,3^ On the one hand, the common description of a trigger event before TGA occurs, implies causality or at least some role in the pathogenesis. On the other hand, the rarity of the phenomenon and the low rate of recurrence (even when the trigger event is likely repeated many times), suggest otherwise.

The most common precipitating factors for TGA found in this study were emotional stress, the Valsalva maneuver and sexual intercourse. As suggested by Lewis [12], transient retrograde venous congestion and venous ischemia to bilateral diencephalic or hippocampal structures could be the common thread connecting all these factors.

This hypothesis suggests that TGA can be triggered by activities that increase venous flow toward the superior vena cava, such as Valsalva maneuvers. In Valsalva, the increased intrathoracic pressure compresses the superior vena cava, obstructing venous flow from the internal jugular vein and transmitting venous back pressure upward toward the brain. It has been shown that patients with TGA are more likely to suffer from internal jugular venous flow reversal (IJVFR) and internal jugular vein valve incompetency (IJVVI) [24–26], supporting this hypothesis. Similarly, emotional states and sexual intercourse can elevate central venous return due to an increase in sympathetic activity [12]. 

### Should we hospitalize TGA patients?

The primary reason for additional testing in TGA is to rule out alternative diagnoses [1]. In order to confirm the diagnosis, other causes of amnesia must be excluded and complete return to baseline mental status must be observed [19]. The ancillary tests performed in our study during hospitalization or as an outpatient did not change the diagnosis. For example, if an abnormal finding such as cardiac arrhythmia (5 patients) or carotid artery stenosis (2 patients) was found, it was considered an incidental finding and did not change the presumed diagnosis to TIA or stroke. Algorithm 1 presents a proposed approach for the management of TGA

**Algorithm 1:**
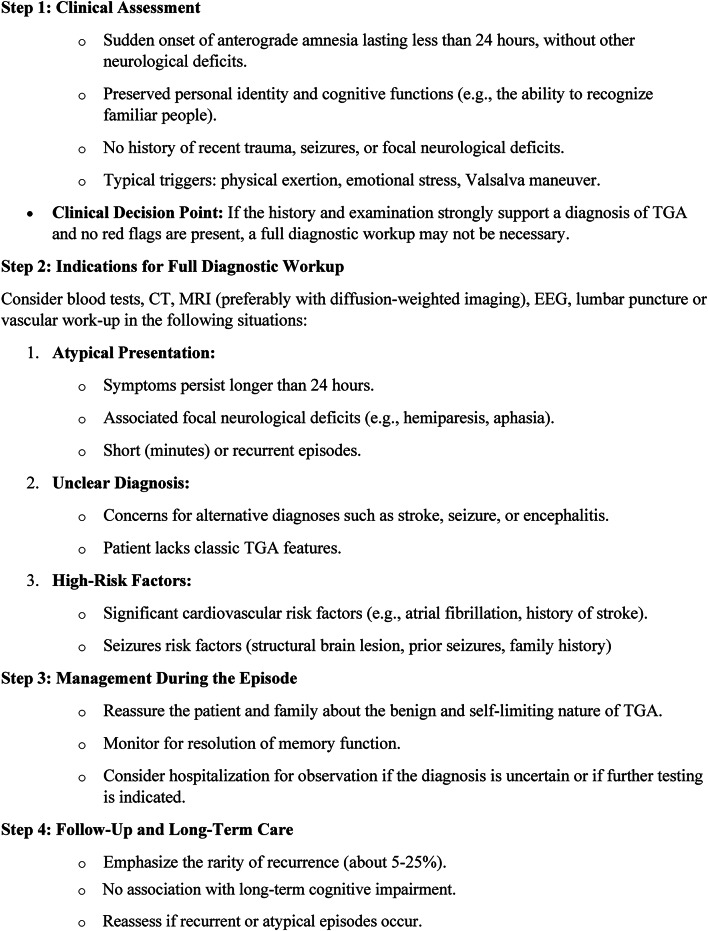
Suggested Algorithm for Diagnosis and Management of TGA

.

In general, the value of EEG after TGA is limited since the majority of results are normal [1]. Similarly, this study found relatively few EEG abnormalities (focal slowing or epileptiform activity). Regarding focal slowings, it is unclear how it relates to TGA. It might represent a true focal dysfunction or might be incidental [27, 28]. 

In terms of epileptiform activity, our findings suggest that a small subgroup of TGA patients may be affected by it. A revision of the EEGs confirmed the presence of epileptiform activity in four patients (three with right temporal activity and one with left temporal activity). Despite the discharge diagnosis being TGA, and even though epileptiform discharges observed on an EEG do not necessarily indicate epilepsy [29], a low dose of an antiepileptic medication was initiated in all cases. Table 3 describes the characteristics of these patients. According to the medical records available to us, we found no evidence of a recurrent TGA or a subsequent suspected seizure in these patients.

**Table 3:** Characteristics and ancillary test results of TGA patients with epileptiform activity on EEG

Characteristics and ancillary test results of TGA patients with epileptiform activity on EEG							
Case	Sex	Age of Onset (years)		Ancillary Tests Results			Medication Started
		Current TGA	Previous TGA	Standard EEG	NCCT	MRI	
1	F	79	–	Right temporal spikes and sharp waves	Right parieto-temporal craniotomy with VP shunt to right lateral ventricle, enephalomalacia in right temporal and frontal lobes	NA	Levetircetam
2	F	72	64	Left TIRDA	Normal	Chronic ischemic changes	Levetircetam
3	F	68	–	Right temporal spikes	Normal	NA	Levetircetam
4	F	74	–	Right temporal spikes	Normal	Chronic ischemic changes	Gabapentin

TGA = transient global amnesia; EEG = electroencephalography; NCCT = non-contrast computed tomography; MRI = magnetic resonance imaging; TIRDA = temporal intermittent rhythmic delta activity; VP = ventriculoperitoneal; NA = not available

### Hippocampal Involvement in TGA

The hippocampus has a key role in short-term memory and consolidation of long-term memory [30]. Hippocampal involvement in TGA is supported by several case series that report one or more punctate lesions in the hippocampus on diffusion-weighted MRI [31–33]. Interestingly, these findings usually appear 12–48 h after the onset of symptoms and resolve after several days (unlike stroke that will appear on MRI much sooner, and typically evolve into a permanent T2 lesion) [32–35]. In our study, MRIs were done late (after discharge) and accordingly did not demonstrate a hippocampal lesion.

Neurons in the hippocampus are known to be selectively vulnerable to metabolic stress, specifically in the setting of ischemia. From a vascular standpoint, vascular density in the hippocampus is a fraction of the vast capillary network in the neocortex. Several studies have mapped the microvasculature of rodents and consistently report the hippocampus has less dense vascularization than other brain regions [36–38]. Diseases that cause even modest reductions in hippocampal blood flow, would likely have a significant impact on neuronal function and memory [39]. It is therefore likely that a multitude of hemodynamic, metabolic, and toxic factors may cause a transient disfunction of the hippocampus that will result clinically as TGA.

### Limitations

First, our study was a single-center study, which may limit the generalizability of the findings to other regions in Israel. Future studies should include multiple medical centers to enhance external validity. Second, due to the retrospective nature of our study, data about certain variables that may be important for understanding the pathogenesis, prognosis, and recurrence rate of TGA are lacking, including personal and family history of migraine, psychiatric disorders, head trauma and dementia [11, 40]. 

## Conclusions

In this study, the epidemiological, clinical, and prognostic characteristics of TGA patients in Israel were evaluated for the first time, filling an important knowledge gap. The findings may facilitate improved patient counseling and physician decision-making by providing a better understanding of disease prevalence, risk factors, and outcomes.

TGA incidence is lower in Israel than in other studies. Cerebrovascular risk factors, migraine and epilepsy, that were suggested as having a pathophysiological role in TGA, were unable to explain this finding. There is also some doubt about the significance of precipitating factors in the development of TGA. Recent studies describing the unique hippocampal vasculature might explain its vulnerability to multitude of hemodynamic, metabolic, and toxic factors. Hopefully, future studies will shed light on the pathogenesis of this condition, and perhaps assist in the understanding of other severe amnestic disorders such as Alzheimer’s disease.

## Data Availability

No datasets were generated or analysed during the current study.
